# Arsenic mineral and compound data as analyzed by X-ray absorption spectroscopy and X-ray diffraction

**DOI:** 10.1016/j.dib.2024.110634

**Published:** 2024-06-19

**Authors:** Valerie A. Schoepfer, Heather E. Jamieson, Matthew B.J. Lindsay

**Affiliations:** a Department of Geological Sciences, University of Saskatchewan, Saskatoon, SK, S7N 5E2, Canada; b Department of Geological Sciences and Geological Engineering, Queen's University, Kingston, ON, K7L 3N6, Canada

**Keywords:** Extended X-ray absorption fine structure, X-ray absorption near edge structure, Mine wastes, Environmental geoscience

## Abstract

Here, we present As K-edge X-ray absorption spectroscopy (XAS) data for 28 arsenic minerals and compounds. These minerals and compounds were obtained from mineral dealers, museum collections, and chemical suppliers, and were positively identified by synchrotron-based powder X-ray diffraction (XRD). All samples were analyzed for both XRD and XAS at the Canadian Light Source synchrotron (Saskatoon, Canada). The As K-edge XAS data were collected in both transmission and fluorescence modes and cover the extended X-ray absorption fine structure (EXAFS) region. Raw XAS data in both modes are provided to support XAS analysis obtained for geological or environmental research. Furthermore, As K-edge EXAFS spectra, the k^3^ weighted oscillatory χ(k) functions, and the Fourier-transforms in χ(R) of these K-edge data are processed and presented graphically. Corresponding XRD data was collected to confirm phase identity. Two-dimensional powder diffraction images were collected against an area detector and integrated to produce line scans. The XRD data were either collected at a wavelength of 0.68866 Å (18 keV) or 0.3497 Å (35.45 keV). Raw, tabulated asc files are available, while the patterns are also presented graphically over a 0-40 °2Θ range or 0-26.5 °2Θ range, respectively. The intent of this dataset is to provide reference XAS spectra to researchers conducting environmental or geological research on As.

Specifications Table

**Table:** 

Subject	Environmental Chemistry
Specific subject area	X-ray absorption spectroscopy and X-ray diffraction for various arsenic minerals and compounds
Data format	Raw, Analyzed
Type of data	Table, Figure
Data collection	A variety of As compounds and minerals were finely ground with a mortar and pestle. All data collection was conducted at the Canadian Light Source synchrotron (Saskatoon, Canada). For XAS, powder was thinly spread between polyimide tape (Kapton) or diluted in boron nitride and packed into a beamline-specific sample holder. Arsenic K-edge EXAFS were collected at the BioXAS-Main wiggler beamline under liquid nitrogen. Samples for XRD were contained within polyimide capillaries and XRD patterns were collected at the CMCF bending magnet [1] or Brockhouse High Energy Wiggler [2] beamlines. The Demeter XAS data package [3] was used for XAS data processing, while XRD data were processed using GSAS-II crystallography software [4].
Data source location	Adamite – Ojuela mine, Mapimi, Durango, Mexico (25.79361, -103.79111). Arsenolite – Purchased. Collected from Cardinal Reward Mine, Black Diamond, Washington, USA (47.30782, -121.94240). Arsenopyrite - Miller Museum Collection, Queen's University. Collected from Rockvale, NSW, Australia (-30.39139, 151.99167). Reference M7629. As metal – CAS: 7440-38-2. As(III)AdsSw – schwertmannite synthesized according to Regenspurg et al. (2004) and adsorbed with approx. 1% As(III). [5] As(III)CopFh – ferrihydrite synthesized according to Schwertmann and Cornell (2000) and coprecipitated with approx. 1% As(III). [5] As(III)CopSw – schwertmannite synthesized according to Regenspurg et al. (2004) and coprecipitated with approx. 1% As(III). [6] As(V)AdsFh – ferrihydrite synthesized according to Schwertmann and Cornell (2000) and adsorbed with approx. 1% As(V). [5] As(V)AdsGt – goethite synthesized according to Schwertmann and Cornell (2000) and adsorbed with approx. 1% As(V). [5] As(V)CopFh – ferrihydrite synthesized according to Schwertmann and Cornell (2000) and coprecipitated with approx. 1% As(V). [5] As(V)CopSw – schwertmannite synthesized according to Regenspurg et al. (2004) and coprecipitated with approx. 1% As(V). [6] As_2_O_3_ – CAS: 1327-53-3. Berzeliite – Personal Collection. Reference 70-C-3. Cobaltite - Miller Museum Collection, Queen's University. Collected from Gowganda, Ontario, Canada (47.64861, -80.76833). Reference M9388. Enargite - University of Saskatchewan Mineral Collection, Saskatoon, Saskatchewan, Canada. Collected from Leonard Mine, Butte, Montana, USA (46.02073, -112.50506). Gersdorffite - Miller Museum Collection, Queen's University. Collected from Dreikönigszug Mine, Mühlbach am Glan, Germany (49.53545, 7.47864). Reference M2179. Hydrous Ferric Arsenate – Synthesized according to Jia et al. (2006) [7] Leiteite – Purchased. Collected from Tsumcorp mine, Tsumeb, Namibia (-19.22704, 17.72764). Lollingite - Miller Museum Collection, Queen's University. Collected from Etta Mine, Keystone, SD, USA (43.88082, -103.41852). Reference M2181.
	Mimetite – Purchased. Collected from Tsumcorp mine, Tsumeb, Namibia (-19.22704, 17.72764). Orpiment - Miller Museum Collection, Queen's University. Collected from Manhattan, Nevada, USA (38.53889, -117.07250). Reference M7651. Realgar - Miller Museum Collection, Queen's University. Collected from Hunan, China (28.1142, 112.9833). Reference M9900. Schneiderhohnite – Purchased. Collected from Galiléia, Minas Gerais, Brazil (18.9992, 41.5385). Scorodite – synthesized according to Rong et al. (2020) [8] Skutterudite - Miller Museum Collection, Queen's University. Collected from Bou Azzer, Morocco (30.5168, 6.9080). Reference M7627. Yukonite - Miller Museum Collection, Queen's University. Collected from Venus Mine, Yukon, Canada (60.02028, -134.63000). Reference M990.
Data accessibility	Repository name: Federated Research Data Repository [12] Data identification number: Direct URL to data: https://doi.org/10.20383/103.0895

## 1 Value of the Data

• Arsenic is a common mine waste contaminant. Substantial research into the identification and speciation of As minerals occurs, but interpretations can be limited without appropriate reference materials.
• Researchers interested in As, especially environmental geochemists, mineral explorationists, and geologists, will benefit by having access to XAS spectra that may not otherwise be available. The data here has all been collected under similar conditions , quality checked, and is available for use without the need to contact multiple authors for data access and scan parameters.
• The intent of this dataset is to provide XAS reference spectra in one location for the identification and characterization of As minerals in complex environments, with all instrument and sample details provided for accurate analysis

## 2 Background

**Fig. 1:**
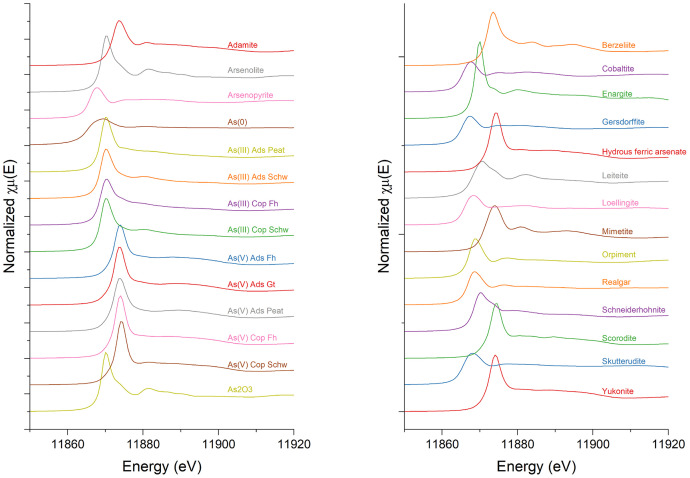
a-b) The As K-edge XAS spectra collected over the XANES region for 28 As-containing reference materials. Spectra are presented over the energy range –20 eV to +100 eV, focused around the As K-edge (11,867 eV) and collected at the BioXAS-Main beamline at the CLS. All spectral data is reported as edge-step normalized absorbance, and were collected either in fluorescence or transmission mode as noted in the associated Athena project file.

**Fig. 2:**
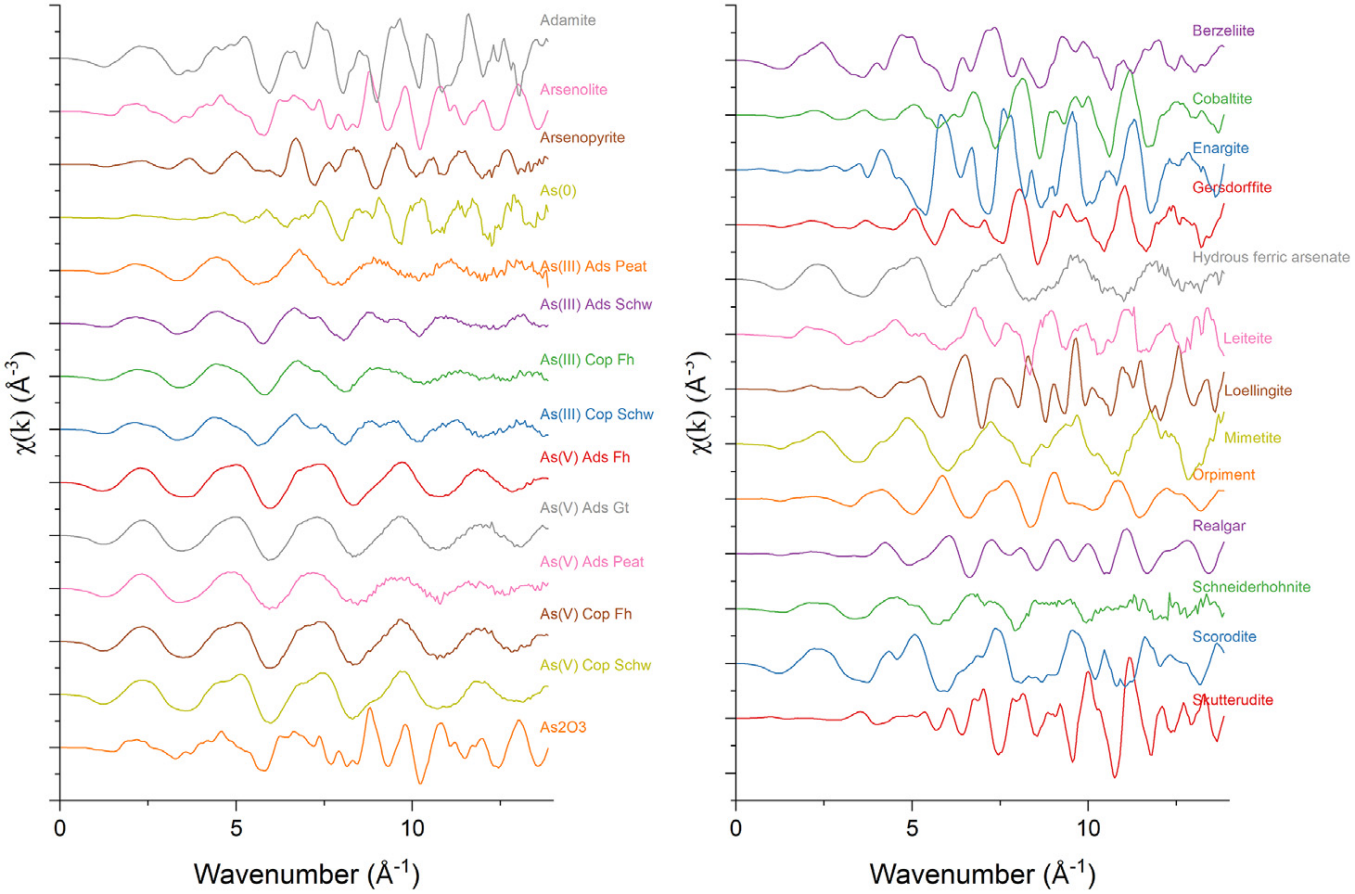
a-b) The k^3^-weighted k-space equivalent of the EXAFS region of each of the 28 references is presented from 0-14 Å^-1^.

**Fig. 3:**
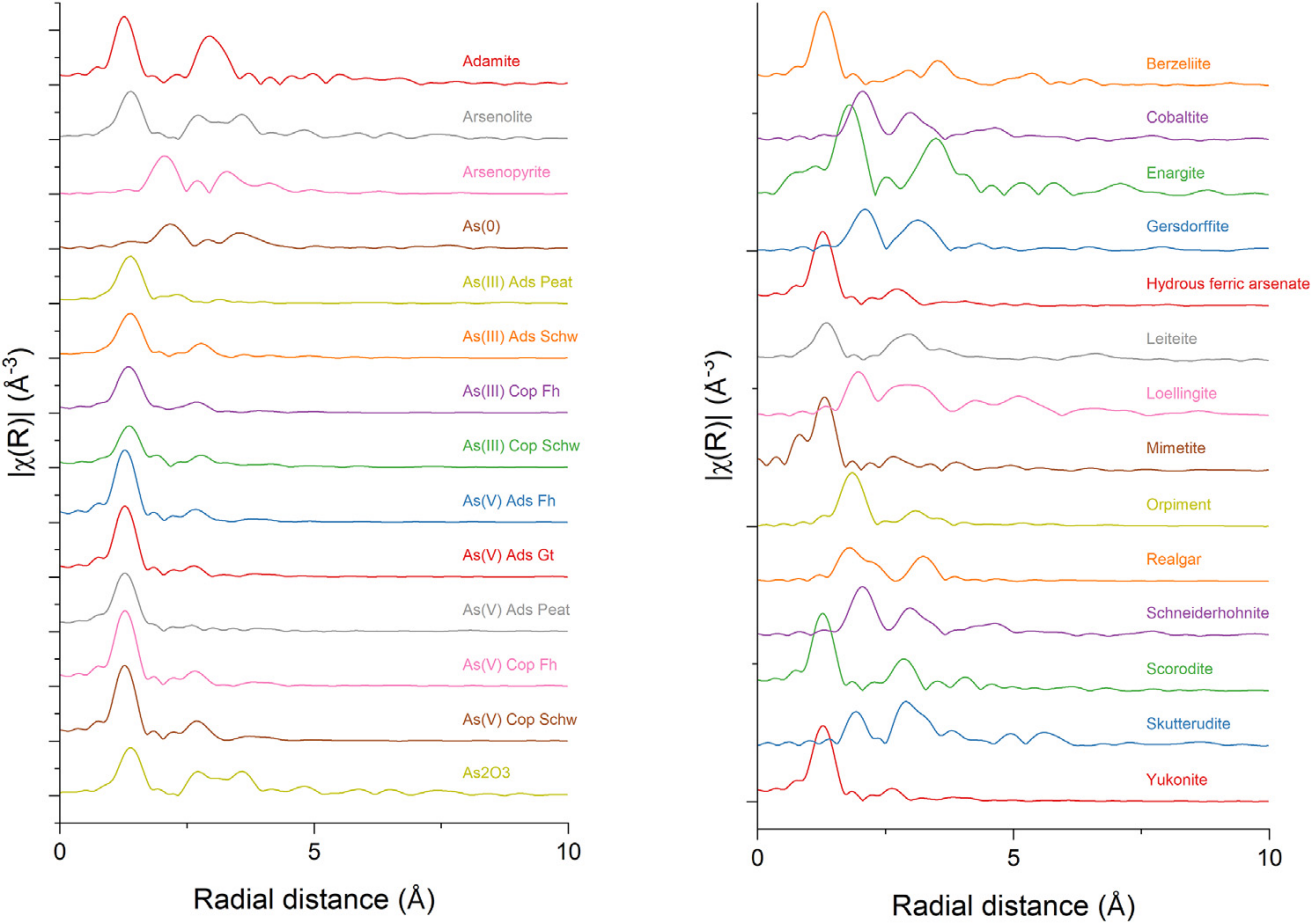
a-b) The Fourier transformed k-space equivalent is presented in R-space for each of the 28 references, where R-space data is shown from 0-10 Å. The Fourier transform was performed over the k-space of 3-11 Å^-1^.

X-ray absorption studies, and in particular, studies focused on the near-edge region, or X-ray Absorption Near Edge Spectroscopy (XANES), often rely on references to identify and interpret raw spectra. In particular, linear combination fitting of this region uses scaled references to identify line shapes and semi-quantify contributions to the spectra. The reliability of associated results are strongly dependent on availability of appropriate reference spectra. However, environmental and mining-related datasets are often complex, and many researchers are either unable to source all relevant references or do not have sufficient synchrotron beamtime to run all potential references. This data set is designed to provide a starting point for environmental and resource extraction-based XAS researchers, where they can test possible spectral lineshapes to streamline and optimize their XAS data collection (Fig. 1, Fig. 2, Fig. 3).

## 3 Data Description

Dataset 1. Energy calibrated As K-edge XAS data collected from -200 eV to +885.6 eV (k=14 Å^-1^) around the As K-edge (11,867 eV) (Fig. 1, Fig. 2, Fig. 3). This folder contains two subfolders and an Athena [3] project file containing all references collected in either transmission or fluorescence mode. The collection mode was chosen for each file individually to best illustrate the peak shapes, i.e. the mode that demonstrated the greatest signal with limited self-absorption and noise. One subfolder contains all raw spectra as collected at the beamline. A second subfolder lists an individual file for each reference as a text-based, xmu file, readable by both Athena [3] and Larch [10]. The xmu files are merged raw files that were energy calibrated but not normalized, in order to preserve the edge jump. The incident energy, measured in the first ionization chamber (I_0_), is listed in the first column and the absorption data of the desired reference compound is presented in the second. The absorption data of an Au(0) reference foil is presented in the third column and was collected between chambers I_1_ and I_2_ for each scan to allow for alignment between scans.

**Fig. 4:**
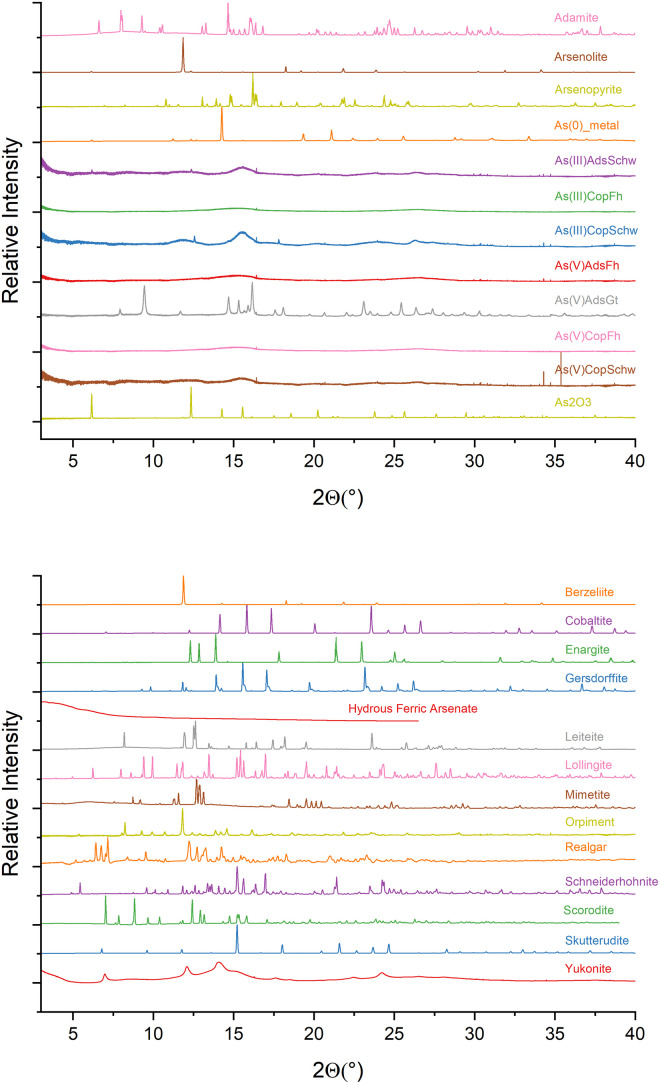
a-b) X-ray diffraction data is presented graphically for all reference materials excluding the peat-based materials. Data was collected at a wavelength of 0.6888 Å (18 keV) at the CMCF beamline at the CLS, with the exception of hydrous ferric arsenate, which was collected at BXDS at the CLS at a wavelength of 0.3497 Å (35.45 keV). All relative intensity data is presented along a °2θ axis.

Dataset 2. X-ray diffraction patterns for reference materials were collected at 18 keV (λ = 0.6888 Å) at CMCF or 35.45 keV (0.3497 Å) at BXDS at the CLS synchrotron (Fig. 4). The CMCF folders contains asc files for each XRD pattern for every available reference from 2-40 °2θ with the exception of hydrous ferric arsenate (HFA). HFA was collected at BXDS from 1.5-26.5 °2θ. Peat-based sample XRD patterns were not collected. The first column in each file presents the collection angle in °2θ and the second column presents the resulting intensity, while the third column is the error term.

## 4 Experimental Design, Materials and Methods

**Reference material preparation** – Selected reference material was isolated and extracted from the matrix material if necessary. Other material was synthesized according to published methods. The resulting sub-samples were finely ground with an agate mortar and pestle (Table 1).

**Table 1:** Details describing the reference materials and minerals prepared in this dataset. Included are the compound's chemical formula, the contained As oxidation state, the ideal mineral space group, crystal system, class, and Nickel-Strunz group (if applicable), and the IMA-CNMNC symbol [9]. A reference to a crystallographic information files is also listed.

Name	Formula	Ox state	Space Group	Crystal System	Nickel-Strunz group	IMA-CNMNC symbol
Adamite^1^	Zn_2_(AsO_4_)(OH)	+5	Pnnm	Orthorhombic	Arsenate	Ad
Arsenolite^2^	As_2_O_3_	+3	Fd $\bar{3}m$	Isometric	Oxide	Aso
Arsenopyrite^3^	FeAsS	-1	P2_1_/b	Monoclinic	Sulfide	Apy
As metal (commercial)^4^	As(0)	0	R $\bar{3}m$	Trigonal	Element	Aso
As(III)AdsSw^5^	As-Fe_8_O_8_(OH)_6_(SO_4_)	+3	P4/m	Tetragonal	Sulfate	Sch
As(III)AdsPeat	As-organic matter	+3				
As(III)CopFh	As-Fe_10_O_14_(OH)_2_	+3		Trigonal	Oxide	Fhy
As(III)CopSw^5^	As-Fe_8_O_8_(OH)_6_(SO_4_)	+3	P4/m	Tetragonal	Sulfate	Sch
As(V)AdsFh	As-Fe_10_O_14_(OH)_2_	+5		Trigonal	Oxide	Fhy
As(V)AdsGt^6^	As-FeOOH	+5	P bnm	Orthorhombic	Oxide	Gth
As(V)AdsPeat	As-organic matter	+5				
As(V)CopFh	As-Fe_10_O_14_(OH)_2_	+5		Trigonal	Oxide	Fhy
As(V)CopSw^5^	As-Fe_8_O_8_(OH)_6_(SO_4_)	+5	P4/m	Tetragonal	Sulfate	Sch
As2O3 (commercial)	As_2_O_3_	+3	R $\bar{3}m$	Trigonal	Element	Aso
Berzeliite^7^	(NaCa_2_)Mg_2_(AsO_4_)_3_	+5	Ia3d	Isometric	Arsenate	Bze
Cobaltite^8^	CoAsS	-1	Pca2_1_	Orthorhombic	Sulfide	Cbt
Enargite^9^	Cu_3_AsS_4_	+3	Pmn2_1_	Orthorhombic	Sulfide	Eng
Gersdorffite^10^	NiAsS	-1	Pa3	Isometric	Sulfide	Gdf
Hydrous Ferric Arsenate	FeAsO_4_*nH_2_O	+5				
Leiteite^11^	Zn(As_2_O_4_)	+3	P2_1_/b	Monoclinic	Oxide	Lt
Löllingite^12^	FeAs_2_	-1	Pnnm	Orthorhombic	Sulfide	Lö
Mimetite^13^	Pb_5_(AsO_4_)_3_Cl	+5	P6_3_/m	Hexagonal	Arsenate	Mim
Orpiment^14^	As_2_S_3_	+3	P21/n	Monoclinic	Sulfide	Orp
Realgar^14^	As_4_S_4_	+3	P21/n	Monoclinic	Sulfide	Rlg
Schneiderhöhnite^15^	Fe_4_As_5_O_13_	+3	P $\bar{1}$	Triclinic	Oxide	Snh
Scorodite^16^	FeAsO_4_*2H_2_O	+5	Pcab	Orthorhombic	Arsenate	Scd
Skutterudite^17^	CoAs_3_	-1	Im3	Isometric	Sulfide	Skt
Yukonite^18^	Ca_3_Fe(AsO_4_)_2_(OH)_3_*5H_2_O	+5		Orthorhombic	Arsenate	Yuk

References

1 Hill, R. J. The Crystal Structure and Infrared Properties of Adamite. American Mineralogist 1976, 61, 979–986.

2 Ballirano, P.; Maras, A. Refinement of the Crystal Structure of Arsenolite, As2O3. Zeitschrift fUr Kristallographie 2002, 217, 177–178.

3 Fuess, H.; Kratz, T.; Topel-Schadt, J.; Miehe, G. Crystal Structure Refinement and Electron Microscopy of Arsenopyrite. Zeitschrift fUr Kristallographie 1987, 179, 335–346.

4 Wyckoff, R. W. G. Crystal Structures; Crystal Structures; Interscience Publishers, 1963.

5 Fernandez-Martinez, A.; Timon, V.; Roman-Ross, G.; Cuello, G. J.; Daniels, J. E.; Ayora, C. The Structure of Schwertmannite, a Nanocrystalline Iron Oxyhydroxysulfate. American Mineralogist 2010, 95 (8–9), 1312–1322. https://doi.org/10.2138/am.2010.3446.

6 Yang, H.; Lu, R.; Downs, R. T.; Costin, G. Goethite, α-FeO(OH), from Single-Crystal Data. Acta Crystallogr E Struct Rep Online 2006, 62 (12), i250–i252. https://doi.org/10.1107/S1600536806047258.

7 Hawthorne, F. C. Refinement of the Crystal Structure of Berzeliite. Acta Crystallogr B Struct Sci 1976, 32 (5), 1581–1583. https://doi.org/10.1107/S0567740876005888.

8 Giese, R. F.; Kerr, P. F. The Crystal Structures of Ordered Adn Disordered Cobaltite. The American Mineralogist 1965, 50.

9 Pauling, L.; Weinbaum, S. The Crystal Structure of Enargite, Cu3AsS4. Zeitschrift für Kristallographie - Crystalline Materials 1934, 88 (1–6), 48–53. https://doi.org/10.1524/zkri.1934.88.1.48.

10 Mauro, D.; Biagioni, C.; Zaccarini, F. New Data on Gersdorffite and Associated Minerals from the Peloritani Mountains (Sicily, Italy). Eur. J. Mineral. 2021, 33 (6), 717–726. https://doi.org/10.5194/ejm-33-717-2021.

11 Ghose, S.; Gupta, P. K. S.; Schlemper, E. O. Leiteite, ZnAs2O4: A Novel Type of Tetrahedral Layer Structure with Arsenite Chains. American Mineralogist 1987, 72, 629–632.

12 Kjekshus, A.; Rakke, T.; Andresen, A. F.; Southern, J. T. Compounds with the Marcasite Type Crystal Structure. IX. Structural Data for FeAs2, FeSe2, NiAs2, NiSb2, and CuSe2. Acta Chem. Scand. 1974, 28a, 996–1000. https://doi.org/10.3891/acta.chem.scand.28a-0996.

13 Dai, Y.; Hughes, J. M. The Crystal Structures of Mimetite and Clinomimetite, Pb5(AsO4)3Cl. Canadian Mineralogist 1991, 29, 369–376.

14 Mullen, D. J.; Nowacki, W. Refinement of the Crystal Structures of Realgar, AsS Annd Orpiment, As2S3. Zeitschrift fUr Kristallographie 1972, 136, 48–65.

15 Cooper, M. A.; Hawthorne, F. C. Refinement of the Crystal Structure of Schneiderhöhnite. Can Mineral 2016, 54 (3), 707–713. https://doi.org/10.3749/canmin.1500102.

16 Kitahama, K.; Kiriyama, R.; Baba, Y. Refinement of the Crystal Structure of Scorodite. Acta Crystallogr B Struct Sci 1975, 31 (1), 322–324. https://doi.org/10.1107/S056774087500266X.

17 Mandel, N.; Donohue, J. The Refinement of the Crystal Structure of Skutterudite, CoAs3. Acta Crystallogr B Struct Crystallogr Cryst Chem 1971, 27 (11), 2288–2289. https://doi.org/10.1107/S0567740871005727.

18 King, G.; Celikin, M.; Gomez, M. A.; Becze, L.; Petkov, V.; Della Ventura, G. Revealing the Structures and Relationships of Ca(II)–Fe(III)–AsO4 Minerals: Arseniosiderite and Yukonite. Environ. Sci.: Nano 2020, 7 (12), 3735–3745. https://doi.org/10.1039/D0EN00503G.

**K-edge XANES and EXAFS analysis** – Less than 20 µg of each finely ground reference material was thinly spread between two layers of polyimide (Kapton) tape. The tape was folded onto itself, and sealed within more polyimide tape. This created two layers of each reference material. Alternatively, between 10-20 µg of the finely ground powder was diluted in boron nitride. These methods were designed to reduce pinhole effects and self-absorption, while allowing sufficient signal transmission and fluorescence.

Arsenic K-edge X-ray absorption spectroscopy (XAS) was performed at the BioXAS-Main wiggler beamline (07ID-2) at the CLS. The BioXAS beamline has an energy resolution of ΔE/E < 1x10^-4^ and a flux of > 1x10^12^ photons/second. The beam focuses to a spot size of approximately 3 x 0.5 mm using Rh-coated toroidal mirrors while a Si(220) double crystal monochromator selects the incident energy. Three downstream ionization chambers and two Ge-32 element fluorescence detectors are included in the beamline setup. A liquid nitrogen cryostat, Soller slits, and Ge fluorescence filters are designed and used to enhance the signal to noise ratio within the resulting As spectra. Spectra were collected in both fluorescence and transmission mode, and the final data mode were chosen based on data quality for each individual sample. All scans ranged from -200 eV below the theoretical As K edge (11,867 eV) to +885.6 eV (k=14 Å^-1^) at 5 eV steps in the pre-edge region (i.e., 11667–11837 eV), 0.5 eV steps in the XANES region (i.e., 11837–11947 eV), and 2.5E+5 eV (k=0.05 Å^-1^) in the EXAFS region (i.e., 11947 eV to +885.6 eV (k=14 Å^-1^)). At least two scans per sample were collected to ensure replicability, reduce noise levels, and eliminate data outliers, while an Au(0) reference foil was collected downstream from the sample between ion chambers I_1_ and I_2_ during each scan for energy calibration and alignment.

**XAS Data preparation and treatment** – Data reduction and preparation was conducted in the Athena program within the Demeter software package [3]. Both fluorescence and transmission data were imported into Athena as µ(E) and corrected for the total flux in I_0_. One mode was chosen individually for each reference based on data quality and the other imports discarded. Each scan was further quality checked and individual scans were discarded for any additional data quality reason. We confirmed replicate scans for each material were aligned and then merged scans. An E_0_ value for each As spectrum and its corresponding Au reference spectrum was selected as the highest point in the main peak of the first derivative. The main peak in the first derivative of the L_III_-edge Au foil was calibrated to 11919 eV [11], and all Au reference foils were aligned. Calibration ensures all As spectra are consistent with external spectra, while alignment ensures internal consistency.

A pre-edge normalization range was visually selected to encompass as large a range as possible while maintaining a flat line. The edge-step normalization range used the typically accurate and automatically selected lower bound, but extended the upper bound to a point near the end of the collected data. This normalization step created normalized spectra from zero to one arbitrary absorbance unit. Text-based data is provided as I_0_ corrected, energy calibrated, and non-normalized µ(E) data, which retains the edge-step associated with each reference material. This method was chosen because concentrations and signal intensity varied per reference material and by analysis mode. However, data normalized to the edge step are presented visually (Fig. 1).

When plotting in χ(k) (Fig. 2) and χ(R) (Fig. 3) space, the R background value for each spectra was individually chosen to be either 0.9 or 1.0 with a k-weight of 3. These R background values were selected visually to minimize the peak in the background function that appeared at k = ∼1, while also not allowing the background function to invert at k < 1. However, the forward Fourier transform k-range was set from 3.0-11.0, which results in the data below k = 3.0 to be ignored. Consequently, the R background function at these low k values was not highly important. The background spline range extended through the dataset from 0-14 k, and corresponded to a spline range in E from 0-748 eV above the As K-edge. There was either no spline clamp or a slight spline clamp in the low energy range, while a strong spline clamp was used in the high energy range to constrain the background function and provide meaningful Fourier transforms. The forward Fourier transform parameters used a k-range from 3.0-11.0 k with a dk of 1 in a Hanning window, while the backwards Fourier transform was from 1-3 with a dR of 0.0 in a Hanning window.

**X-ray diffraction** – Finely ground powder samples were loaded into 0.5 mm inner diameter polyimide (Kapton) capillaries around 2.2 cm in length. These capillaries were sealed on both ends with ethyl cyanoacrylate adhesive.

Hydrous ferric arsenate was analysed for powder X-ray diffraction (XRD) at BXDS (04ID-2) at the CLS [2], where the sample was directly loaded onto a multi-sample holder. Here, the capillary was quickly jiggled back and forth for approximately 60 seconds in front of a 35.45 keV (λ = 0.3497 Å) beam with a flux of approximately 1.5x10^11^ photons/second. The BXDS Perkin Elmer area detector was positioned approximately 570 mm from the sample and 64 one-second scans were collected to result in a 2Θ range of 1.5-26.5°.

All other references were analysed at the CMCF-BM beamline (08B1-1) [1], where capillaries were loaded into magnetic Unipuck stubs, placed on a SSRL cassette, mounted by a robotic arm to the goniometer using the Stanford Automated Mounting system, and rotated during the 30-60 second exposure to the 18 keV (λ = 0.6888 Å) beam at room temperature. The resulting data ranged from 2-40° 2θ. The CMCF Pilatus3 S 6M X-ray detector was at a distance of 350 mm and used an aperture of 200 µm to collect up to six 30 second scans. The flux at CMCF was also approximately 1.5x10^11^ photons/second.

The resulting 2-D concentric images were processed using the program GSAS-II [4], where instrument parameters, including the exact detector distance, were first calibrated using a LaB_6_ or Ni(0) calibrant. Following calibration, sample files were imported into GSAS-II and an image of an empty capillary exposed to the beam for the same duration as the associated sample was subtracted. Images were summed with equal weighting and scans were integrated to convert the two-dimensional image to a line scan. Data were saved as powder Topas XYE files, and manually converted to asc files. Plotted data is normalized from 0-1 (Fig. 4).

## Limitations

None.

## Ethics Statement

The authors declare that the manuscript adheres to the ethics in publishing standards for Data in Brief. The current work does not involve human subjects, animal experiments, or any data collected from social media platforms.

## CRediT authorship contribution statement

**Valerie A. Schoepfer:** Formal analysis, Validation, Investigation, Data curation, Writing – original draft, Visualization. **Heather E. Jamieson:** Resources, Writing – review & editing. **Matthew B.J. Lindsay:** Resources, Validation, Writing – review & editing, Supervision, Funding acquisition.

## Data Availability

X -ray absorption spectroscopy and X-ray diffraction data for arsenic minerals and compounds: Datasets and Supplementary Materials (Original data) (Federated Research Data Repository).
